# Radiocarpal joint stiffness following surgical treatment for distal radius fractures: the incidence and associated factors

**DOI:** 10.1186/s13018-020-01857-6

**Published:** 2020-08-11

**Authors:** Lingde Kong, Yajie Zhai, Zuzhuo Zhang, Jian Lu, Bing Zhang, Dehu Tian

**Affiliations:** 1 Department of Orthopedics, The Third Hospital of Hebei Medical University, 139 Ziqiang Road, Shijiazhuang, 050051 Hebei People’s Republic of China; 2 Department of Radiology, The Third Hospital of Hebei Medical University, Shijiazhuang, 050051 Hebei People’s Republic of China

**Keywords:** Wrist, Distal radius, Stiffness, Prognosis, Prevalence

## Abstract

**Background:**

Postoperative radiocarpal joint stiffness (RJS) is common in patients with distal radius fractures (DRFs). The purpose of this study was to record the incidence of RJS and to determine potential risk factors that may be associated with it.

**Methods:**

We retrospectively included a series of patients who suffered from DRFs and underwent volar plate fixation. Patients’ basic data, radiographic data, and postoperative data were collected. The incidence of RJS during follow-up was recorded, and both univariate analyses and multivariate logistic regression were used to determine factors associated with it.

**Results:**

A total of 119 patients were included in this study. After surgical procedures, there were 42 (35.3%) patients with RJS and 77 (64.7%) patients without. The incidence of RJS after surgical treatment is 35.3%. Multivariate analysis showed that intra-articular fracture (OR, 1.43; 95% CI, 1.13–1.81), pre-operative severe swelling (OR, 1.35; 95% CI, 1.05–1.74), post-operative unsatisfied volar tile (OR, 1.38; 95% CI, 1.01–1.89), and improper rehabilitation exercise (OR, 1.72; 95% CI, 1.18–2.51) were correlated with the incidence of RJS during follow-up.

**Conclusions:**

Patients with intra-articular fracture, pre-operative severe swelling, post-operative unsatisfied volar tile, and improper rehabilitation exercise were factors associated with the incidence of wrist stiffness. Preoperative risk notification and postoperative precautions are necessary for relevant patients.

## Background

Distal radius fractures (DRFs) are the most common fractures of the upper limb and the most common fractures overall in individuals younger than 75 years [1, 2]. As a common injury, it account for approximately one sixth of all fractures in emergency departments [3, 4], and both conservative treatment and surgical procedure have been used to treat it [5–7]. For unstable fractures, surgery can provide improved radiologic parameters and better functional outcomes at an early stage of treatment. As the most common form of internal fixation, volar locking plate is widely used in the treatment of unstable DRFs. Patients could have an earlier return to normal activities with the reliable fixation of volar locking plate [8–10].

The main goal of treatment is obtaining sufficient pain-free motion, allowing early return to activities and minimizing the risk for future degenerative changes or disability. However, even with surgical treatment, radiocarpal joint stiffness (RJS) is a common postoperative complication following DRFs, which greatly influence patients’ normal function [11, 12]. Previous studies have investigated many postoperative complications following DRFs, such as tendon spontaneous rupture [13, 14], loss of reduction [15, 16], injury of triangular fibrocartilage complex [17], or wrist pain [18, 19]. To the best of our knowledge, no studies have been reported about postoperative RJS.

In this study, we tried to fill some of these gaps by analyzing DRFs patients who underwent volar locking plate fixation. The aim is to record the incidence of postoperative RJS in patients at 6 months follow-up and to identify possible risk factors that may be associated with the RJS.

## Materials and methods

### Patient population

This study reviewed a series of patients who suffered from DRFs between January 2015 and February 2018. The inclusion criteria were adult patients with closed DRFs confirmed by x-ray test or computed tomography (CT) scan and underwent surgical treatment with volar locking plate fixation. Patients were excluded if they had previous DRFs, open fractures, bilateral DRFs, ipsilateral upper extremity injuries, or concomitant arterial or nerve injury. This study was approved by the Research and Ethics Committee of the Third Hospital of Hebei Medical University, and all patients gave written informed consent for their information to be stored in the database of this hospital and used for medical research.

### Treatment and follow-up

All patients were performed open reduction and internal fixation after brachial plexus or general anesthesia. The locking plate is applied through an incision over the volar aspect of the wrist, and no dorsal approach was used. A standard flexor carpi radialis approach to the distal radius was applied. After exposure of the distal radius, reduction procedure was performed first, and volar locking plate with or without Kirschner wire was used to fix it. All operations were performed by three senior surgeons. The details of the surgical approach, the type of plate, and the number and configuration of screws were decided by surgeons. Some surgeons used a cast/splint after surgery, but the fixed angle stability provided by the locking plate is generally sufficient to allow early controlled range of movement exercises. The use or otherwise of a cast/splint was also at the discretion of surgeons. Finger, elbow, and shoulder exercises were started at the first day after surgery.

Routine follow-up was performed postoperatively at 2, 4, and 6 weeks and 3 and 6 months. At each visit, patients were asked to perform x-ray tests. At the 6 months follow-up, the range of radiocarpal joint movement was measured and recorded.

### Data collection

Associated factors were evaluated from three aspects, which are basic data, radiographic data, and postoperative data. The basic data were collected from medical record, such as age, gender, body mass index (BMI), habits, concomitant diseases, preoperative swelling, and time from injury to surgery. Preoperative swelling was assessed on the first day of hospitalization. If the wrist is swelling than the contralateral side but the skin texture can be recognized, the swelling was considered to be slight. If the skin texture cannot be recognized or blisters occurred, the swelling was considered to be severe.

**Fig. 1:**
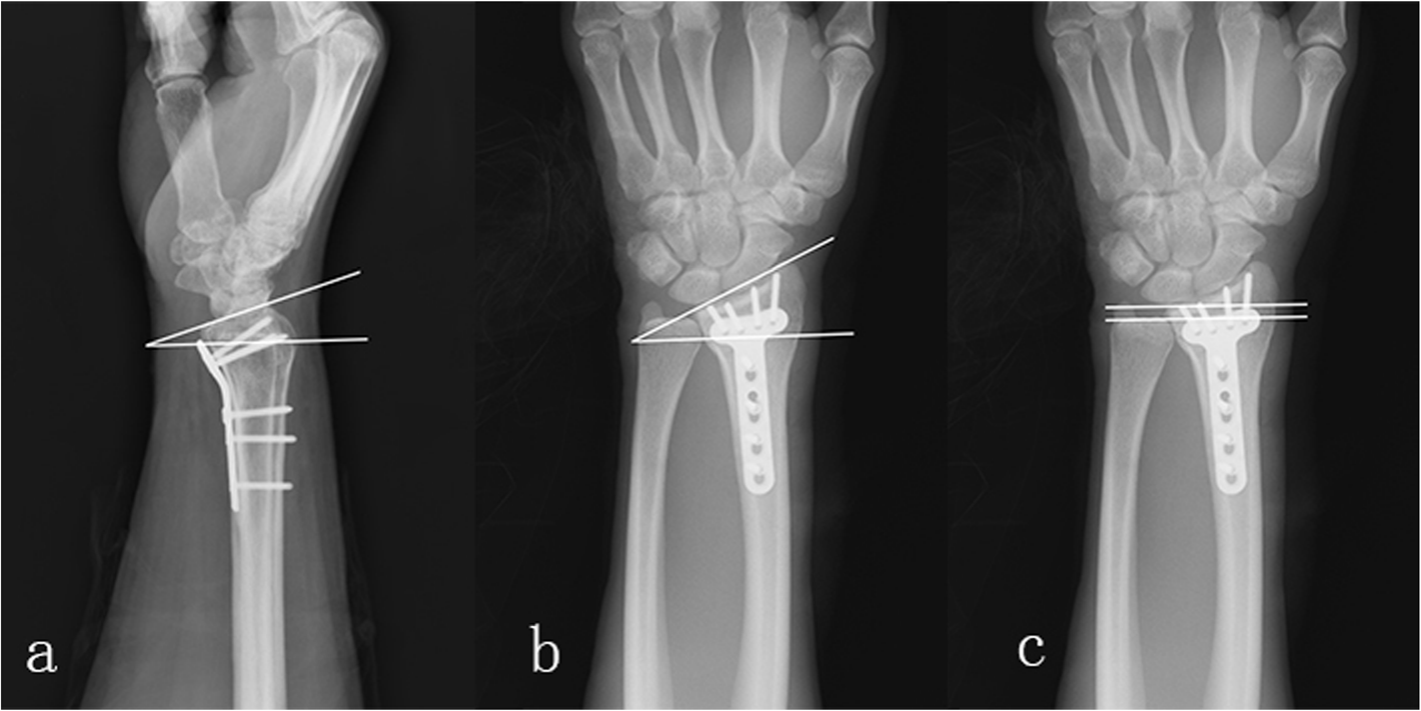
Postoperative x-ray film showing the measurement of radiographic parameters. **a** Volar tilt was the angle between the line along the distal radial articular surface and the line perpendicular to the longitudinal axis of the radius at the joint margin. **b** Radial inclination was the angle formed between the long axis of the radius and a line drawn from the distal tip of the radial styloid to the ulnar corner of the lunate fossa. **c** We identified the long axis of the radius and drew a line perpendicular to this, extending through the ulnar-most corner of the lunate fossa. Ulnar variance was the distance between this line, and the distal-most point of the ulnar dome was recorded as the ulnar variance

There are five parameters in radiographic data. Fracture type and concomitant ulnar styloid process fracture were collected from preoperative radiograph, and volar tilt, radial inclination, and ulnar variance were measured from the radiograph at 6 months follow-up. Fracture type was divided into intra-articular fracture and extra-articular fracture according to whether the fracture line crosses articular surface. Volar tilt was measured on lateral radiograph by determining the angle between the line along the distal radial articular surface and the line perpendicular to the longitudinal axis of the radius at the joint margin. Radial inclination was measured on a anteroposterior (AP) radiograph by determining the angle formed between the long axis of the radius and a line drawn from the distal tip of the radial styloid to the ulnar corner of the lunate fossa. Ulnar variance was measured on a AP radiograph using the method of perpendiculars. We identified the long axis of the radius and drew a line perpendicular to this, extending through the ulnar-most corner of the lunate fossa. The distance between this line and the distal-most point of the ulnar dome was recorded as the ulnar variance [20]. The way to measure these radiographic parameters is shown in Fig. 1. Wrist exercise advice was given by surgeons according to the status of fracture healing, which is a standard post-operative rehabilitation protocol including six wrist movements at a certain frequency. At the end of follow-up, patient compliance was asked about. Patients who followed the advice were considered to perform proper wrist rehabilitation exercise; otherwise, they were considered to perform improper wrist rehabilitation exercise.

The radiocarpal joint movement after surgery was assessed with a goniometer. As flexion and extension of wrist are two most frequently used movements in our everyday life and can compensate for other wrist movements in most activities, we use the range of flexion-extension motion to assess the existence of RJS. The RJS was considered to present if the range of flexion-extension motion was no more than half of that in the contralateral side.

### Statistical analysis

All statistical analyses were performed using the Statistical Package for the Social Sciences (SPSS), version 18.0. Variables were presented as a mean with standard deviation for continuous variables and with frequencies and percentages for categorical variables. The independent sample *t* test or Mann-Whitney *U* test was used for numerical data, and Fisher’s exact test was used to identify differences in frequency of nominal variables between groups. After univariate analyses, variables found to be potentially associated with the RJS (*P* < 0.20) were included in the multivariate logistic regression models. *P* values less than 0.05 were considered to be statistically significant.

## Results

A total of 119 patients who underwent distal radius volar locking plate fixation were included in the current study. Among these patients, 30 (25.2%) were male, and 89 (74.8%) were female. The mean age at the time of surgery was 55.4 ± 9.6. Preoperative swelling was considered to be slight in 44 patients (37.0%) and was severe in 75 patients (63.0%). Extra-articular fractures were seen in 23 patients (19.3%), and intra-articular fractures were in 96 patients (80.7%). During surgery, 58 fractures (48.7%) were fixed with plate only, and the other 61 fractures (51.3%) were fixed with plate and Kirschner wire. Forty-one patients (34.5%) had assisted cast or splint fixation after surgery, and the other 78 patients (65.5%) had not. There were four superficial incision infection (3.4%), two apparent loss of reduction (1.7%), and one intra-articular screw penetration (0.8%). Forty-two patients (35.3%) were considered to be RJS, and the other 77 patients (64.7%) were not.

In the analysis of the association between patients’ basic data (Table 1) and radiographic data (Table 2) as well as postoperative data (Table 3) and the incidence of RJS, we found that age (*P* = 0.04), preoperative swelling (*P* = 0.01), types of internal fixation (*P* = 0.18), fracture type (*P* = 0.05), post-operative volar tile (*P* = 0.01), and improper rehabilitation exercise (*P* = 0.02) were potential risk factors, while gender, BMI, history of smoking or alcohol, diabetes mellitus, osteoporosis, dominant hand, time from injury to operation, ulnar styloid process fracture, post-operative radial inclination, post-operative ulnar variance, assisted cast or splint fixation, postoperative infection, or removal of internal fixation was not (*P* ≥ 0.20).

**Table 1:** The comparison of basic data in patients with and without radiocarpal joint stiffness

Variables	With stiffness (*n* = 42)	Without stiffness (*n* = 77)	*P* value
Age (years)	57.9 ± 10.8	54.1 ± 9.1	0.04
Gender			
Male	11	19	0.86
Female	31	58	
BMI (kg/m^2^)	23.9 ± 4.9	24.3 ± 4.2	0.64
Smoker			
Yes	8	14	0.91
No	34	63	
Drinker			
Yes	9	11	0.32
No	33	66	
Diabetics			
Yes	13	23	0.90
No	29	54	
Osteoporosis			
Yes	22	39	0.86
No	20	38	
Dominant hand			
Yes	24	40	0.59
No	18	37	
Time from injury to operation (days)	3.5 ± 2.3	3.2 ± 2.5	
Preoperative swelling			
Slight	9	35	0.01
Severe	33	42	
Internal fixation			
Plate only	17	41	0.18
Plate and Kirschner wire	25	36	

*BMI* body mass index

**Table 2:** The comparison of radiographic data in patients with and without radiocarpal joint stiffness

Variables	With stiffness (*n* = 42)	Without stiffness (*n* = 77)	*P* value
Fracture type			
Extra-articular	4	19	0.05
Intra-articular	38	58	
Ulnar styloid process fracture			
Yes	18	31	0.78
No	24	46	
Post-operative radial inclination (degree)	19.3 ± 3.5	20.1 ± 3.6	0.24
Post-operative volar tilt (degree)	6.4 ± 1.4	7.2 ± 1.8	0.01
Post-operative ulnar variance (mm)	0.3 ± 0.2	0.4 ± 0.2	0.37

**Table 3:** The comparison of postoperative data in patients with and without radiocarpal joint stiffness

Variables	With stiffness (*n* = 42)	Without stiffness (*n* = 77)	*P* value
Assisted cast or splint fixation			
Yes	13	28	0.55
No	29	49	
Postoperative infection			
Yes	1	3	0.66
No	41	74	
Proper rehabilitation exercise			
Yes	20	54	0.02
No	22	23	
Removal of internal fixation			
Yes	25	40	0.43
No	17	37	

In the further multivariate logistic regression analysis, intra-articular fracture (OR, 1.43; 95% CI, 1.13–1.81), pre-operative severe swelling (OR, 1.35; 95% CI, 1.05–1.74), post-operative unsatisfied volar tile (OR, 1.38; 95% CI, 1.01–1.89), and improper rehabilitation exercise (OR, 1.72; 95% CI, 1.18–2.51) were demonstrated to be associated with the incidence of RJS during follow-up (Table 4).

**Table 4:** Multivariate logistic regression analysis of factors associated with radiocarpal joint stiffness

	*P* value	Odds Ratio	95% CI
Elder age (≥ 60 years)	0.26	1.17	0.90–1.53
Internal fixation (plate and Kirschner wire)	0.37	1.16	0.83–1.61
Fracture type (intra-articular)	0.02	1.43	1.13–1.81
Pre-operative swelling (severe)	0.03	1.35	1.05–1.74
Post-operative volar tilt (≤ 7°)	0.03	1.38	1.01–1.89
Improper rehabilitation exercise	0.01	1.72	1.18–2.51

*CI* confidence interval

## Discussion

Identification of associated factors for RJS may help surgeons to find patients at the greatest risk for it and to adjust their monitoring and follow-up decisions. In this study, we only reviewed patients underwent volar locking plate fixation and revealed that the incidence of RJS was 35.3% after 6 months follow-up. Patients with intra-articular fracture, pre-operative severe swelling, post-operative unsatisfied volar tile, and improper rehabilitation exercise were demonstrated to be correlated with the incidence of RJS during follow-up. Therefore, risk notification before surgery and precautions after surgery are necessary for these patients.

Our result showed that intra-articular fracture was associated with a diminished flexion/extension arc. Intra-articular fracture usually involves step-offs and gaps, which can result in residual articular incongruence even after surgical treatment. Karnezis et al. [21] analyzed different types of fractures and concluded that the presence of postoperative articular incongruity of 1 mm or more is associated with persisting loss of wrist dorsiflexion and wrist joint dysfunction at 1 year following injury. Previous studies also showed that persistent intra-articular incongruity may cause a 9-fold increased risk of radiological osteoarthritis [22, 23]. We think that restoration of the articular surface and maintenance of joint congruity were essential for good postoperative wrist movement.

Dorsal angulation, radial inclination, and radial shortening were three parameters used to assess the distal radius deformity [24]. Surgeons usually strive for an anatomic reduction, yet obtaining the anticipated reduction may be very hard or even impossible in some cases. Based on the study published by Grewal and MacDermid [25], alignment was considered acceptable if the residual dorsal angulation was ≤ 10°, radial inclination was ≥ 15°, and radial shortening with positive ulnar variance was < 3 mm. In the assessment of radiographic parameters, post-operative unsatisfied volar tile was demonstrated to be associated with the incidence of RJS independently. According to previous studies, dorsal angulation of radiocarpal joint surface worsens functional outcome considerably when it exceeds 20° [26, 27], and each 10° of dorsal angulation diminishes volar flexion by 3° [22]. Dorsal angulation exceeding 20° caused a 6 and 8° loss of volar flexion after the injury respectively [28]. Although anatomic reduction is the ideal goal of treatment, restoration of radial inclination and radial shortening are not as critical as volar tilt for wrist dorsiflexion movement.

The mechanism of injury is another important prognostic indicator. High-energy creating fractures usually cause damage to around ligaments and soft tissue at the same time. The severity of soft tissue damage cannot be observed directly and is hard to measured, but can be reflected indirectly by limb swelling. In the current study, preoperative wrist swelling was demonstrated to be associated with RJS. Although a correlation was seen, we cannot confirm a causal relationship between them.

Postoperative rehabilitation exercise is important for orthopedic surgery. A home exercise was considered to be as effective as formal physical therapy [29], but controversy still exists in other problems, for example, the timing of postoperative immobilization. A prospective randomized study compared early wrist exercise (i.e., within 2 weeks of surgery) with late wrist exercise (i.e., 6 weeks) in DRFs patients treated with volar plate fixation, and concluded that no significant differences were identified with respect to the average flexion-extension arc of the injured wrist at 3 or 6 months follow-up [30]. In our study, the lack of exercise within 3 months is common in these patients with improper rehabilitation exercise. We inferred that early or late exercise may not significantly affect the RJS, but the lack of exercise dose. Doctors’ advice and patients’ compliance during follow-up are essential for RJS prevention.

Strengths of this study are the strict inclusion criteria and the homogeneity of patients. However, there are several limitations. First of all, we only studied a diminished flexion/extension arc in the assessment of RJS, and ulnar/radial deviation arc of motion was not investigated. Secondly, though wrist motion is important in everyday life, the impact of RJS on functional outcome cannot be determined based on current study. Thirdly, all results were come from patients after surgical treatment; thus, we cannot apply the results to all DRFs patients. Finally, the follow-up time is relatively short, which is only 6 months. Wrist degenerative changes or osteoarthritis may occur in long-term follow-up and should be recorded in further studies.

## Conclusions

In summary, about one third of RDFs patients would show RJS after volar locking plate fixation at the 6 months follow-up. Intra-articular fracture, preoperative severe swelling, post-operative unsatisfied volar tile, and improper rehabilitation exercise were factors that may be associated with the incidence of RJS during follow-up. Therefore, risk notification before surgery and precautions after surgery are necessary for these patients.

## Data Availability

Not applicable.
